# Course of self-reported symptoms of 342 outpatients receiving medium- versus long-term psychodynamic psychotherapy

**DOI:** 10.1186/s13030-016-0074-4

**Published:** 2016-07-29

**Authors:** S. Nolte, L. Erdur, H. F. Fischer, M. Rose, B. Palmowski

**Affiliations:** 1 Department of Psychosomatic Medicine, Center for Internal Medicine and Dermatology, Charité - Universitätsmedizin Berlin, Berlin, Germany; 2 Population Health Strategic Research Centre, School of Health and Social Development, Deakin University, Melbourne, VIC Australia; 3 Institute for Social Medicine, Epidemiology and Health Economics, Charité - Universitätsmedizin Berlin, Berlin, Germany; 4 Quantitative Health Sciences, Outcomes Measurement Science, University of Massachusetts Medical School, Worcester, MA USA; 5 Private Practice Dr Bernhard Palmowski, Berlin, Germany

**Keywords:** Psychodynamic psychotherapy, Psychosomatic medicine, Longitudinal data, Symptom rating, Self-report data, Outcomes assessment, Observational study

## Abstract

**Background:**

The course of self-reported symptoms during medium- versus long-term psychodynamic psychotherapy has rarely been documented for outpatient settings. This observational study describes routine practice of ambulatory treatment in Germany and explores self-reported symptoms of a broad patient sample undergoing one (medium-term) versus two years (long-term) of psychodynamic psychotherapy.

**Methods:**

Over four and a half years, longitudinal self-report symptom data were collected from 342 outpatients as part of a standardized documentation system. Self-report data were compared between patients receiving either medium-term or long-term psychodynamic psychotherapy.

**Results:**

Routine care significantly decreased disease burden as reported by patients by small to medium effect sizes (ES) for depression (ES = 0.58), anxiety (ES = 0.49), obsessive-compulsive disorder (ES = 0.54), somatoform disorder (ES = 0.32), eating disorder (ES = 0.38). The majority of patients completed treatment after one year and showed medium-size changes. For a subgroup of patients with depressive and/or obsessive-compulsive disorder symptoms for whom two years of therapy were deemed necessary, additional benefits were reported during the second year of treatment (ES = 0.61 and ES  0.47, respectively).

**Conclusions:**

Our findings suggest that both medium- and long-term psychodynamic psychotherapy decrease self-reported disease burden of patients with depression, anxiety, obsessive-compulsive, somatoform and/or eating disorders. For a subgroup of patients, additional benefits were gained in the second year of treatment.

## Background

The burden of mental illness is increasing worldwide [1], with much debate that current estimations are even underestimates of the true burden of mental and behavioral disorders [2–4]. Besides psychopharmacological treatment, psychotherapy is widely applied in Germany, and physicians specialized in psychosomatic medicine apply this type of treatment in approximately 65 % of their patients [5]. In Germany, the provision of psychotherapy according to approved guidelines is covered by the public health insurance system. This includes cognitive behavioral therapy (CBT) and psychodynamic psychotherapy (including psychoanalyses). Both therapeutic approaches have been proven to be effective and efficacious [6, 7]. However, these results are mostly derived from the so-called “gold standard” in therapy research, the randomized controlled trials (RCT) design. RCTs assure that effects can be directly attributed to the specific treatment by means of standardization and by randomly assigning patients to treatment or control groups [8]. Apart from ethical issues, Westen *et al.* [9] expressed critique on the application of RCTs in psychotherapy research, pointing out the mismatch between the experimental setting and psychotherapy practice in reality. One aspect is that in RCTs there are often manualized therapies for specific disorders with an average of 20–30 sessions, whereas in clinical reality outpatient psychotherapy is regularly provided over the course of about 50–70 sessions depending on the disorder [9]. Hence, evidence is needed for outcomes from psychotherapy that takes place in a real-world setting rather than in an artificial research environment.

Further, research to date has usually focused on outcomes of short-term therapy, which generally found effectiveness for a range of patients. However, research also suggests that short-term psychotherapy may fail specific subgroups of patients, for example, those affected by complex mental disorders [10–13] for whom long-term treatment may be necessary. As evidence from research into long-term approaches is scarce, Leichsenring and Rabung [14] undertook a meta-analysis on outcomes of long-term psychodynamic psychotherapy from 23 studies. Their meta-analysis suggests superiority of long-term over short-term treatment, with large effect sizes for aforementioned subgroups of patients who do not sufficiently benefit from short-term interventions [14]. While this meta-analysis is an important contribution to the field, it again also emphasizes the lack of research in this area as only few studies could be included in the analysis. Further, samples tended to be rather small, with a range of different outcomes assessed via mostly different instruments. Most importantly, included studies were frequently carried out in inpatient settings and/or in artificial study environments such as control group situations. While the latter is the acknowledged gold standard, and it is a type of research design that is crucial to ensure internal validity that allows for causal inference as detailed above [15, 16], these settings often lack clinical representativeness [14]. It therefore remains that—even after such great effort to collate data on outcomes from long-term therapy—evidence from real-world psychodynamic psychotherapy in outpatient settings, i.e. where most treatment takes place, remains scarce [17].

The aim of the present observational study is to give an overview of outcomes from psychodynamic psychotherapy and to describe the course of patient-reported symptom severity over medium-term (one year) versus long-term (two years) psychodynamic psychotherapy in an ambulatory care setting. The paper addresses two research questions: 1) Does the severity of patient-reported symptoms change under medium- or long-term psychodynamic psychotherapy? 2) Does a second year of therapy lead to additional improvements in self-reported symptoms, i.e. is psychodynamic psychotherapy beyond one year justified for certain subgroups of patients?

## Methods

### Study design and setting

Between June 2008 and December 2012 routine data were collected from adult patients receiving either one or two years of psychodynamic psychotherapy who were treated in 13 outpatient clinics run by practice-based Specialists for Psychosomatic Medicine and Psychotherapy. Clinics were located in either Northern or Eastern Germany, of which about half of the clinics were based in Germany’s capital of Berlin.

We included consecutive patients who presented at participating clinics as described above, with routine data collected once per year. Patients had to be 18 years or older, and be able to read and understand German. Of above patient cohort, we excluded those who had provided data within the last 15 months before closure of the database as it could not be ruled out that these patients were still in active psychotherapy. As these patients potentially would not yet have received the full dose of treatment, it was important to exclude these to receive valid estimates from the data that were collected. Applying above criteria led to a final sample size of *n =* 342. Of these, 271 (79 %) patients received one year of psychodynamic psychotherapy and 71 (21 %) patients received two years of treatment.

### Intervention

In Germany, psychotherapy (cognitive-behavioral therapy and psychodynamic psychotherapy) is covered by the public health insurance system. That is, costs for short-term (up to 25 sessions), medium-term (25–50 sessions) as well as long-term therapies (maximum 80–100 sessions for psychodynamic psychotherapy; up to 300 sessions for psychoanalyses) are reimbursed [18].

Psychodynamic psychotherapy embraces a comprehensive curative approach that—in addition to purely focusing on symptom-oriented relief—leads to a stronger psychosocial capacity to cope with future challenges of life. It focuses on revealing and verbalizing emotions. This particularly applies to contradictory emotional attitudes and efforts, and the potential for conflict to be discovered which is clarified in everyday life as well as within treatment. Habitual avoidance strategies are to be experienced, understood, and changed. Recurring patterns in interpersonal relationships as well as hidden fantasies and motives are identified and explored systematically. The life-historical process of formation of reactions and attitudes is examined and integrated in the personality of the patient. This applies to interpersonal relationships outside as well as to the therapeutic alliance itself, with the latter being the essential therapeutic agent [19, 20]. Psychodynamic psychotherapy consists of individual and group sessions with both carried out according to aforementioned principles. While individual sessions may be scheduled in irregular intervals, group sessions mostly occur regularly, that is, usually once per week. Groups are generally mixed in terms of sex, age, social status, and symptoms.

In the current study, all patients received routine psychodynamic psychotherapy as described above, including both individual doctor-patient sessions and group therapy with or without pharmacological treatment. As the majority of patients received a mix of both approaches, i.e. one-on-one sessions and group therapy, we applied the following definition for patients to be considered as mostly receiving “group therapy”: >60 % of the sessions received were group therapy sessions with >10 group therapy sessions attended. Patients not fulfilling these criteria were considered as patients receiving “one-on-one”. Although treatment intensity and quantity differed between patients, with the schedule ranging from low-frequency sessions once a week or once every two weeks up to high frequency arrangements with two to three sessions per week, it needs to be noted that treatment provided by Specialists for Psychosomatic Medicine and Psychotherapy is regulated in Germany. That is, the therapists are obliged to follow treatment methods that have been scientifically approved and are included in the catalogues of services of the German statutory insurance funds. The continuing medical education includes supervised short-, medium- and long-term outpatient psychotherapy, regular meetings with colleagues for therapeutic self-awareness sessions, and inpatient clinical training. Hence, all therapists who provide psychodynamic psychotherapy in the psychosomatic medicine setting are qualified in short-, medium- and long-term psychodynamic treatment.

### Data documentation system

At collaborating institutions, patient data were collected routinely using the Documentation System in Psychosomatic medicine (DSP). Patients filled in questionnaires on 1) socio-demographic data including use of healthcare services and 2) the ICD-10-Symptom-Rating (ISR) scale [21], whereas therapists filled in questionnaires on 3) diagnoses based on the German ICD-classification system ICD-10-GM [22] and 4) use of other health care services [17].

In view of socio-demographic data both sex and age were available from patients’ health records. Education, employment status, nationality, and relationship status were assessed via patient self-report. For this paper, education was grouped following the categorization as recommended by the German Federal Ministry of Health and used in many federally funded projects [23].

The ISR, developed in Germany, assesses patient-reported symptoms [21] based on chapter F, ICD-10-GM [22]. The ISR aims at assessing presence as well as severity of self-reported symptoms. It consists of 29 items, of which 17 form the five subscales depressive (4 items), anxiety (4 items), obsessive-compulsive (3 items), somatoform (3 items), and eating disorder (3 items) symptoms. The remaining 12 items can be used as a screening instrument for single syndromes. In this paper we use the former five subscales only as these measure distinct aspects of mental health [24]. The ISR items are scored on a 5-point Likert scale ranging from 0 (‘Does not apply’) to 4 (‘Extremely applies’). Subscale scores are calculated by forming the average of respective responses per individual subscale; hence, scores in a given subscale range from 0 (no symptoms) to 4 (maximum amount of self-reported symptoms). The psychometric properties of the ISR have been shown to be very strong, with evidence that it is largely robust against potential confounding effects such as response shift when measuring change over time [25]. Further details on the ISR can be found elsewhere [21].

### Statistical analyses

All analyses are based on the most prevalent diagnoses in ambulatory psychosomatic care. The following diagnoses based on ICD-10-GM [22] were selected: depressive disorder (F32.- to F39), anxiety disorder (F40.- to F41.-), obsessive-compulsive disorder (F42.-), somatoform disorder (F45.-), eating disorder and obesity (F50.-; E66.-). Obesity was included because of its increasing relevance and lack of a corresponding diagnosis in ICD-10-GM chapter F.

Demographic data assessed at baseline (T_0_) are presented descriptively, comparing patients receiving one year versus those receiving two years of psychodynamic psychotherapy, using chi-square and *t*-test statistics for independent samples. Further, the most frequently reported diagnoses based on the ICD-10-GM as documented by respective clinician and the number of comorbidities are compared between groups, with special emphasis on those diagnoses of chapter F with a corresponding subscale in the ISR. Symptom severity, as measured by the ISR at baseline (T_0_), was compared between groups using robust ANOVA Brown-Forsythe [26].

For longitudinal analyses, both raw mean ISR scores and effect sizes (ES) were compared a) longitudinally within each group and b) between groups, i.e. patients who received one year versus those who received two years of psychodynamic psychotherapy. For the comparison of raw mean ISR scores, one-sample t-tests were calculated. For the comparison of effects sizes, ES were calculated by taking the ISR difference score divided by the standard deviation (SD) at baseline [27]. Interpretation of ES followed Cohen’s suggestion for interpreting effect size d, with ES ~ 0.2 considered a small, ES ~ 0.5 a medium, ES ~ 0.8 a large effect [27]. All analyses were carried out using IBM SPSS Statistics 22.0®.

### Ethical considerations and data protection

As self-reported symptom data were acquired as part of routine patient assessment, i.e. the aforementioned DSP. Each patient was informed about the study and had to provide written informed consent at baseline which included the right to decline participation in this study. Less than 1 % declined participation.

All data were pseudonymized with unique identification numbers assigned to each individual patient to ensure linkage of longitudinal data. Those responsible for the data cleaning, analyses, interpretation of results, and writing up of the manuscript (SN, LE, FF, MR) did not have access to patient names at any time but were blinded to any possible identification of individual patients.

## Results

Baseline characteristics of patients with one year of psychodynamic psychotherapy (*n =* 271) compared to those with two years of therapy (*n =* 71) are presented in Table 1. Overall, more women (65 %) compared with men (35 %) received psychodynamic psychotherapy. Mean age of patients was 45.5 (SD = 12.0) years; about 40 % stated to be single. As shown in Table 2, less than 10 % of patients were diagnosed with a single disease based on ICD-10-GM but the majority of patients had one or more comorbidities, with an average of 4.27 (SD = 2.46) documented diagnoses. When comparing patients receiving medium- with those receiving long-term therapy, the latter group showed a higher mean of diagnoses, with 4.12 (SD = 2.40) diagnoses for patients in medium-term and 4.85 (SD = 2.63) diagnoses for patients in long-term therapy (*p* = .027). In view of chapter F of ICD-10-GM, the most common clinical diagnosis was depression (61 %) followed by anxiety (37 %) and somatoform disorder (28 %). Patients with somatoform disorders were more likely to receive long-term psychotherapy (χ^2^ = 4.997, *p* = .025), while there were no statistically significant differences between patients receiving one year versus those receiving two years of therapy for other diagnostic groups within chapter F. There were small, albeit not statistically significant, trends towards an increase of the proportion of women and those with a clinical diagnosis of depression, obsessive-compulsive disorder, and somatoform disorder in the group of patients receiving long-term psychodynamic psychotherapy. As for diagnoses based on other chapters of the ICD-10-GM, the most frequently documented diagnoses were back pain (M54.-) recorded 61 times, sleep disorders (G47.-) recorded 43 times, and migraines and headaches (G43.-, G44.-) recorded 39 times (Table 2).

**Table 1:** Baseline characteristics of patients treated by German practice-based Specialists for Psychosomatic Medicine; total sample (*n =* 342), comparison of patients with one year (*n =* 271) versus those with two years of treatment (*n =* 71)

	Total sample		Treatment 1 year		Treatment 2 years	
	*n =* 342		*n =* 271		*n =* 71	
	n	%	n	%	n	%
Gender						
Male	125	36.5	104	38.4	21	29.6
Female	217	63.5	167	61.6	50	70.4
Age in years						
Mean (SD)	45.5 (12.0)		45.9 (12.1)		44.0 (11.4)	
Range	22–81		22–81		26–75	
Age groups						
up to 30 years	36	10.5	28	10.3	8	11.3
30–39 years	77	22.5	57	21.0	20	28.2
40–49 years	92	26.9	74	27.3	18	25.4
50–59 years	99	28.9	80	29.5	19	26.8
60+ years	38	11.1	32	11.8	6	8.5
Education^a^						
Year 9 or less	37	10.8	30	11.2	7	9.9
Year 9 w/ prof. training	48	14.0	37	13.8	11	15.5
Year 10 w/ prof. training	98	28.7	80	29.7	18	25.4
Year 13, w/ or w/out prof. training	84	24.6	64	23.8	20	28.2
Year 13, w/ university	73	21.3	58	21.6	15	21.1
Employment status						
Full-time	142	41.5	112	41.3	30	42.3
Part-time	61	17.8	52	19.2	9	12.7
Unemployed	48	14.0	35	12.9	13	18.3
Other	91	26.6	72	26.6	19	26.8
Relationship status						
No partner	134	39.2	105	38.7	29	40.8
Married	115	33.6	92	33.9	23	32.4
Partner, not married	93	27.2	74	27.3	19	26.8

^a^Education following categorization as applied in the German national KiGGS Survey (Lange *et al.*, 2007)

*Significant group differences at *p <* .05 level (chi-square tests; for age: *t*-test statistic for independent samples)

**Table 2:** Overview of reported diagnoses at baseline as documented by clinician and overview of most frequently recorded diagnoses by chapter ICD-10-GM; total sample (*n =* 342), comparison of patients with one year (*n =* 271) versus those with two years of treatment (*n =* 71)

	Total sample		Treatment 1 year		Treatment 2 years	
	*n =* 342		*n =* 271		*n =* 71	
	n	%	n	%	n	%
Diagnoses (ICD-10-GM)						
Number of diagnoses (mean, SD)*	4.27 (2.46)		4.12 (2.40)		4.85 (2.63)	
1 diagnosis	31	9.1	26	9.6	5	7.0
2–5 diagnoses	222	64.9	182	67.2	40	56.3
6–10 diagnoses	89	26.0	63	23.2	26	36.6
Chapter F (Mental and behavioral disorders)^a^						
Depression (F32.- to F39)	208	60.8	159	58.7	49	69.0
Anxiety (F40.- to F41.-)	128	37.4	100	36.9	28	39.4
Obsessive-compulsive disorder (F42.-)	43	12.6	30	11.1	13	18.3
Somatoform disorder (F45.-)*	94	27.5	67	24.7	27	38.0
Eating disorder (F50.-; E66.-)	68	19.9	51	18.8	17	23.9
Presence of ≥1 other diagnoses chapter F	206	60.2	163	60.1	43	60.6
Chapter E (Endocrine, nutritional, metabolic)						
Presence of ≥1 diagnoses chapter E	58	17.0	45	16.6	13	18.3
Chapter G (Diseases of nervous system)						
Presence of ≥1 diagnoses chapter G	83	24.3	67	24.7	16	22.5
Chapter M (Musculoskeletal system)						
Presence of ≥1 diagnoses chapter M	78	22.8	61	22.5	17	23.9
Other (predominantly chapters I, K and R)						
Presence of ≥1 further diagnoses*	182	53.2	136	50.2	46	64.8

^a^As patients were frequently diagnosed with more than one clinical diagnosis (ICD-10-GM), the sum of diagnoses is larger than the total sample size because of co-morbidities

*Significant group differences at p < .05 level (chi-square tests; for mean number of diagnoses: *t*-test statistic for independent samples)

Regarding doctor-patient encounters per year, an average of 34.1 (SD = 21.2) scheduled meetings were recorded for the total sample (*n =* 342). While there was a trend towards those receiving two years of psychodynamic psychotherapy having more doctor-patient encounters, particularly in their first year of therapy (M = 38.2, SD = 23.8), compared to those with one year of treatment (M = 33.0, SD = 20.3), differences were not statistically significant. As shown in Table 3, there was also no statistically significant difference with regard to group versus one-on-one treatment between patients receiving medium- versus long-term psychodynamic psychotherapy (medium-term: *n =* 207 (76 %) one-on-one; long-term: *n =* 49 (70 %) one-on-one).

**Table 3:** Distribution of types of treatment (one-on-one versus group therapy) for diagnostic subgroups; total sample (*n =* 342), and patients receiving medium-term (*n =* 271) versus patients receiving long-term psychodynamic psychotherapy (*n =* 71)

	Total sample^b^		Treatment 1 year		Treatment 2 years	
	*n =* 342		*n =* 271		*n =* 71	
	n	%	n	%	n	%
One-on-One						
Total Sample	256	74.9	207	76.4	49	69.0
Depressive Disorder^a^ (F32.- to F39)	148	71.2	115	72.3	33	67.3
Anxiety Disorder (F40.- to F41.-)	97	75.8	77	77.0	20	71.4
Obsessive Compulsive Disorder (F42.-)	14	32.6	11	36.7	3	23.1
Somatoform Disorder (F45.-)	62	66.0	45	67.2	17	63.0
Eating Disorder (F50.-; E66.-)	57	83.8	42	82.4	15	88.2
Group Therapy						
Total Sample	86	25.1	64	23.6	22	31.0
Depressive Disorder (F32.- to F39)	60	28.8	44	27.7	16	32.7
Anxiety Disorder (F40.- to F41.-)	31	24.2	23	23.0	8	28.6
Obsessive Compulsive Disorder (F42.-)	29	67.4	19	63.3	10	76.9
Somatoform Disorder (F45.-)	32	34.0	22	32.8	10	37.0
Eating Disorder (F50.-; E66.-)	11	16.2	9	17.6	2	11.8

^a^As patients were frequently diagnosed with more than one clinical diagnosis (ICD-10-GM), the sum of diagnoses is larger than the total sample size because of co-morbidities

^b^The distribution between one-on-one and group therapy did not differ significantly between patients receiving one versus those receiving two years of treatment

In view of self-reported symptoms at baseline (T_0_) as measured by the ICD-10-Symptom-Rating scale, no significant differences were observed between patients receiving medium- versus those receiving long-term psychodynamic psychotherapy. Patients’ mean ISR scores were comparable across self-reported depressive, anxiety, obsessive-compulsive, somatoform, and eating disorder symptoms (Table 4). When selecting only those patients who had a positive clinical diagnosis corresponding to the symptoms as measured by the ISR, self-reported symptoms at baseline again did not differ between the two groups receiving different lengths of treatment (data not shown). For longitudinal assessment, symptom severity as shown by raw mean ISR scores decreased significantly between T_0_ and follow-up (T_1_) in both groups across all five ISR domains, with the exception of eating disorder in the group of patients with two years of treatment. This group reported ISR scores at T_1_ that were worse compared to those they had reported at T_0_. For the group of patients with two years of treatment, a third measurement time point of self-reported symptoms was available. As shown in Table 4 at the bottom of the rightmost column, compared to their ISR at T_0_ as well as their first follow-up scores (T_1_), this group of patients showed significant improvements across all five ISR domains at their last measurement time point (T_2_), reflected in a decrease in self-reported ISR scores.

**Table 4:** ICD-Symptom-Rating (ISR) scores at baseline (T_0_), 1-year follow-up (T_1_), 2-year follow-up (T_2_); total sample and comparison of patients with one year of treatment (*n =* 271) versus those with two years of treatment (*n =* 71)

ISR scale^a^	Total sample	Treatment 1 year	Treatment 2 years
	*n =* 342	*n =* 271	*n =* 71
	mean (SD)	mean (SD)	mean (SD)
Baseline (T_0_)			
Depressive symptoms	2.04 (0.97)	2.07 (0.98)	1.96 (0.93)
Anxiety symptoms	1.86 (1.08)	1.84 (1.08)	1.91 (1.06)
Obsessive-compulsive disorder symptoms	1.52 (1.09)	1.56 (1.09)	1.35 (1.07)
Somatoform symptoms	0.85 (1.03)	0.84 (1.04)	0.89 (0.99)
Eating disorder symptoms	0.84 (1.07)	0.84 (1.09)	0.85 (1.01)
1-year follow-up (T_1_)			
Depressive symptoms	1.56 (0.95)*	1.55 (0.94)*	1.56 (0.96)*
Anxiety symptoms	1.54 (1.03)*	1.51 (1.03)*	1.65 (1.00)*
Obsessive-compulsive disorder symptoms	1.22 (0.99)*	1.20 (0.99)*	1.30 (0.99)*
Somatoform symptoms	0.67 (0.82)*	0.63 (0.79)*	0.79 (0.93)*
Eating disorder symptoms	0.81 (0.98)*	0.78 (0.98)*	0.91 (0.98)*
2-year follow-up (T_2_)			
Depressive symptoms			1.31 (0.87)*
Anxiety symptoms			1.47 (0.93)*
Obsessive-compulsive disorder symptoms			1.05 (1.02)*
Somatoform symptoms			0.55 (0.74)*
Eating disorder symptoms			0.80 (0.76)*

Note, there were no significant differences between the group with one year compared with the group with two years of treatment regardless of which follow-up scores were compared

^a^ISR items are scored from 0 to 4; subscale scores are calculated by forming the average of respective responses; hence, mean scores in a given subscale range from 0 (no self-reported symptoms) to 4 (maximum amount of self-reported symptoms)

*Significant differences at *p <* .05 level (robust ANOVA, Brown-Forsythe) between respective Baseline, 1-year, and 2-year follow-up scores

Longitudinal data were further investigated by calculating effect sizes. These analyses again suggest that symptom burden decreased significantly as a result of psychodynamic psychotherapy, with a comparable course of self-reported symptoms for both groups, i.e. the group of patients with medium-term and the group of patients with long-term psychodynamic psychotherapy reported a decrease in self-reported symptoms over time. That is, at the end of respective treatment (T_1_ for medium-term, T_2_ for long-term therapy) the total sample of patients with a positive clinical diagnosis of the disorders investigated in this study reported improvements ranging from ES = 0.32 (95 % CI: 0.18,0.47) for somatoform disorder symptoms to ES = 0.58 (95 % CI: 0.45,0.70) for depressive symptoms (Table 5, first column). In contrast, subgroup analyses showed that groups’ effect sizes differed when basing ES on the comparison of T_0_ with T_1_ scores, i.e. a point in time when patients with medium-term therapy had completed treatment, while those who received long-term therapy were still in active treatment (Table 5, second versus third column). Significant group differences were found for self-reported somatoform and eating disorder symptoms. Marked, albeit not statistically significant, differences were also seen for depressive and obsessive-compulsive disorder symptoms. Finally, when basing each group’s ES on respective maximum follow-up ISR score, i.e. a point in time when all patients had completed treatment, however, differences between the two groups were not statistically significant anymore (Table 5, second versus fourth column).

**Table 5:** ISR difference scores (based on effect sizes [ES]^a^) of patients with a positive clinical diagnosis (depression, anxiety, obsessive-compulsive, somatoform and/or eating disorders) matched to according self-report ISR scores; total sample (*n =* 342) and comparison of patients with one year of treatment (*n =* 271) versus those with two years of treatment (*n =* 71)

ISR scale	Total sample	Treatment over 1 year	Treatment over 2 years	
	ES after completion of treatment	ES after completion of treatment	ES before completion of treatment	ES after completion of treatment
	ES (T_max_-T_0_)^b^	ES (T_1_-T_0_)	ES (T_1_-T_0_)^c^	ES (T_2_-T_0_)^d^
	ES (95 % CI)	ES (95 % CI)	ES (95 % CI)	ES (95 % CI)
Depressive symptoms	Positive clinical diagnosis of depressive disorder			
	*n =* 208 0.58 (0.44,0.71)	*n =* 159 0.56 (0.41,0.72)	*n =* 49 0.37 (0.09,0.65)	*n =* 49 0.61 (0.33,0.90)
Anxiety symptoms	Positive clinical diagnosis of anxiety disorder			
	*n =* 128 0.45 (0.29,0.62)	*n =* 100 0.44 (0.26,0.63)	*n =* 28 0.44 (0.12,0.76)	*n =* 28 0.49 (0.11,0.87)
Obsessive-compulsive disorder symptoms	Positive clinical diagnosis of obsessive-compulsive disorder			
	*n =* 43 0.54 (0.31,0.77)	*n =* 30 0.57 (0.30,0.84)	*n =* 13 0.23 (–0.24,0.71)	*n =* 13 0.47 (–0.05,0.99)
Somatoform symptoms	Positive clinical diagnosis of somatoform disorder			
	*n =* 94 0.31 (0.15,0.48)	*n =* 67 0.39 (0.18,0.60)*	*n =* 27 0.04 (–0.16,0.23)*	*n =* 27 0.13 (–0.10,0.37)
Eating disorder symptoms	Positive clinical diagnosis of eating disorder			
	*n =* 68 0.29 (0.07,0.52)	*n =* 51 0.35 (0.10,0.60)*	*n =* 17 –0.21 (–0.58,0.16)*	*n =* 17 0.13 (–0.41,0.67)

^a^Effect sizes (ES) interpreted as small (ES ~ 0.2), medium (ES ~ 0.5) or large (ES ~ 0.8) effects (Cohen, [27])

^b^ES based on T_max_ (maximum), i.e. ES are based on the latest follow-up ISR data available, i.e. T_1_ (*n =* 271) and T_2_ (*n =* 71), respectively

^c^ES based on (T_1_-T_0_), i.e. this group’s first follow-up score

^d^ES based on (T_2_-T_0_), i.e. this group’s final follow-up score

*Significant differences at *p <* .05 level (robust ANOVA, Brown-Forsythe for the comparison of patients with one year of treatment [*n =* 271] versus those with two years of treatment [*n =* 71], with the latter group having two follow-up scores, i.e. at T_1_ and at T_2_); patients with a positive diagnosis of respective symptom only

**Fig. 1:**
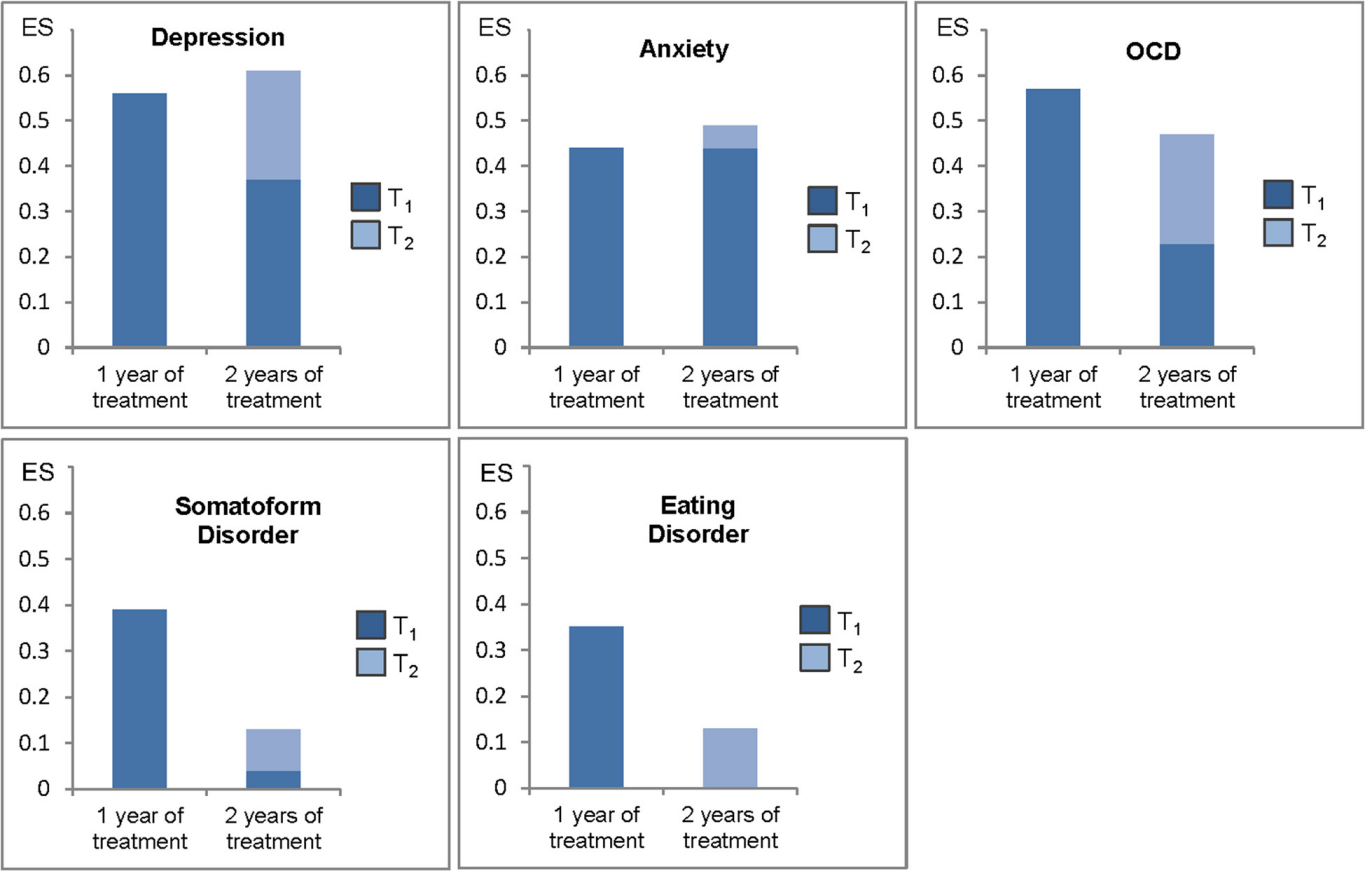
Effect sizes (ES) of patients receiving medium-term psychosomatic treatment (1 year) compared to those with long-term treatment (2 years) across five ISR domains

For depressive, anxiety, and obsessive-compulsive disorder symptoms, ES across groups were of medium size. In contrast, ES for somatoform disorder symptoms were small, particularly for the group receiving long-term therapy. For eating disorder symptoms, the group receiving medium-term psychodynamic psychotherapy almost reached medium-size ES, while effects were substantially, although not significantly, smaller for the group in long-term therapy (Fig. 1).

## Discussion

This observational study was aimed at investigating the course of self-reported symptoms as reported by patients receiving medium-term (1 year) compared to those receiving long-term (2 years) psychodynamic psychotherapy, with a treatment regime following established methods typical for German psychosomatic outpatient care [28].

Baseline data suggest that patients who receive medium- or long-term psychodynamic psychotherapy not only suffer from a wide range of mental and behavioral disorders but also have a high prevalence of additional disorders, with the vast majority of patients having both mental and somatic comorbidities. Patients were characterized by medium age (mean age 45 years), with about two thirds being women. It is noteworthy that hardly any statistically significant differences were found between patients receiving medium-term compared to patients receiving long-term psychodynamic psychotherapy. That is, overall neither baseline characteristics nor patients’ self-reported symptoms as measured by the ISR were associated with treatment length. The only trends were that the group of patients receiving long-term therapy regularly showed a higher prevalence of a range of diagnoses, with statistically significant differences for mean number of diagnoses with those in long-term therapy showing a higher mean number of diagnoses; however, group differences by diagnostic groups were mostly not statistically significant. Further, the female-to-male ratio was also somewhat higher in the group of patients in long-term therapy. While it is conceivable that compliance to psychotherapy among women is higher compared to men, this possible explanation cannot be substantiated following two meta-analyses on discontinuation of psychotherapy where no such connection was found [29, 30].

Regarding longitudinal assessment, our data suggest that self-reported symptom burden decreased significantly over time. When expressed in terms of effect sizes, small to medium ES across all main health domains were found, with largest ES observed for depressive, anxiety, and obsessive-compulsive disorder symptoms. Further, we found that the subgroup of patients that required two years of therapy (*n =* 71) showed rather small ES at T_1_, i.e. at a point in time when their treatment was still ongoing. That is, ES were consistently smaller compared to the group of patients who had completed treatment at that point (*n =* 271). At T_2_, i.e. a point in time when the former group was considered as having completed their therapy as well, differences in ES between the two groups were not statistically significant anymore. Even though this is not a classic dose-effect-model as investigated by others [10], our findings suggest that long-term psychodynamic psychotherapy seems justified for this particular subgroup as many of these patients had not reached saturation at T_1_. However, when comparing the five diagnostic groups explored in this study, this is only true for patients with depressive or obsessive-compulsive disorders. Patients with anxiety disorders did not show further improvements in the additional year of treatment, and the small group of patients with somatoform or eating disorders—who did not show any improvement in self-reported symptoms within the first year of treatment—did not in their second year either.

The comparison of our results to other studies in this area is somewhat difficult. To our knowledge, there are only few studies in the field of psychodynamic psychotherapy that explore treatment outcomes from medium- or long-term therapy in an outpatient setting such as the one presented herein. An overview of long-term psychodynamic psychotherapy is provided in aforementioned meta-analysis [14], which includes studies reporting treatment outcomes that were assessed in the context of an observational study design similar to ours. However, studies that were included have limited comparability to our study sample. Those that seem largely comparable showed similar effect sizes [31–33].

Our study provides an overview of the course of self-reported symptoms collected as part of a widely implemented routine health outcomes assessment for psychosomatic care in Germany. The main finding is that a clinically meaningful reduction of self-reported disease burden was demonstrated over time of treatment for both medium- and long-term psychodynamic psychotherapy. This finding highlights the strengths of psychodynamic psychotherapy. This type of therapy does not primarily focus on symptom relief but it aims at helping the patient to get a more comprehensive view of her-/himself and to reveal unconscious motives in her/his behavioral patterns and interpersonal relationships. It is assumed that achieving these aims leads to a decrease in symptoms. This decrease in self-reported symptoms was also observed in our study. However, despite the small to medium alleviation of patient-reported symptom burden as found in our observational study, it remains unclear whether the specific effect is attributable to the intervention alone. To address the comparative effectiveness of ambulatory psychodynamic psychotherapy in a real-world setting, a cohort study would be necessary to attribute benefits to the treatment as provided, including appropriate control groups, to ensure internal validity of our results [15, 16]. Moreover, patient-reported outcomes assessments should also include further variables, such as the change of functional impairments or impact in daily life under psychotherapy. Nevertheless, we believe that in our study we were able to provide a good overview of the course of self-reported symptoms obtained from real-world medium- versus long-term psychodynamic psychotherapy under routine care conditions.

## Conclusion

Our paper is based on data collected under routine care conditions. That is, data were obtained from a clinical population that is likely to be representative of current psychosomatic care in Germany, which has not yet received sufficient attention in the literature. In summary, patients from a wide range of backgrounds undergo psychodynamic psychotherapy in the German ambulatory psychosomatic care setting, with those receiving medium-term or long-term psychodynamic psychotherapy reporting a small to medium size decrease in self-reported symptom burden across a range of health domains. Our analyses further show that patients who are in treatment for two years report small benefits within the first year of therapy but gain additional benefits during the second year of therapy. This supports the notion that this subgroup may need more time to respond to psychodynamic psychotherapy. Overall, our study shows that psychodynamic psychotherapy improves symptom severity in a wide range of mental disorders.
